# Effects of propofol on colon cancer metastasis through STAT3/HOTAIR axis by activating WIF‐1 and suppressing Wnt pathway

**DOI:** 10.1002/cam4.2840

**Published:** 2020-01-17

**Authors:** Yun‐Fei Zhang, Chang‐Sheng Li, Yi Zhou, Xi‐Hua Lu

**Affiliations:** ^1^ Department of Anesthesiology Affiliated Cancer Hospital of Zhengzhou University, Henan Cancer Hospital Zhengzhou P.R. China

**Keywords:** colon cancer, lncRNA HOTAIR, propofol, STAT3, Wnt signaling

## Abstract

**Background:**

In the present study, we aim to investigate the potential role of propofol in the tumor progression of colon cancer.

**Methods:**

Human colon cancer cell lines were cultured and exposed with 8 μg/mL propofol. RNA interference was performed to silence the expression of HOTAIR or STAT3 to explore their biological functions in colon cancer. Cell apoptosis and invasion were assessed using flow cytometry and transwell assays, respectively. Quantitative real‐time PCR, western blot, and immunohistochemistry were subjected to measure the expression patterns of HOTAIR, STAT3, Wnt signaling factors, and epithelial‐mesenchymal transition‐related markers, respectively. Besides, nude mice were transplanted with colon cancer cells for further exploration. Tumor formation, volume, and weight were evaluated to validate the in vitro findings.

**Results:**

Propofol treatment promoted cell apoptosis and inhibited cell invasion in colon cancer cells, while the effects were reversed by HOTAIR overexpression. Additionally, STAT3 positively regulated HOTAIR expression, which was also negatively modulated by propofol. Moreover, STAT3 and HOTAIR were shown to independently regulate colon cancer cell apoptosis and invasion. Furthermore, HOTAIR could stimulate Wnt signaling pathway via inhibiting WIF‐1 expression and upregulating β‐catenin expression, which was also demonstrated by in vivo study.

**Conclusion:**

Taken together, the current study demonstrated that propofol exerts the inhibition on cell invasion and promotion on cell apoptosis through regulating STAT3/HOTAIR by activating WIF‐1 and suppressing Wnt pathway, indicating that propofol might serve as a therapeutic role for colon cancer patients in the future.

## Graphical abstract

Propofol regulates the colon cancer cell apoptosis and invasion via down‐regulation of lncRNA HOTAIR. STAT3 could regulate the expression level of HOTAIR. STAT3 and HOTAIR synergistically regulates the colon cancer cell apoptosis and invasion.

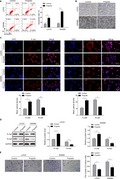

## 1 INTRODUCTION

Colon cancer is one of the most common forms of cancer.^1^ Currently, surgical treatment remains the mainstay curative treatment for nonmetastasized colon cancer,^2^ while for metastatic colon cancer, chemotherapy combinations and targeted therapies are commonly used.^3^ The targeted therapies for colon cancer mainly target epithelial growth factor receptor (EGFR) or vascular endothelial growth factor (VEGF).^1^ For anti‐EGFR therapies, both cetuximab and panitumumab showed objective response rate of ~10% when used as monotherapy for metastatic colon cancer,^4^, ^5^ while the resistance to the anti‐EGFR therapies was later demonstrated to be mainly due to tumor somatic mutations in KRAS gene.^6^, ^7^ However, even in patients with wild‐type KRAS gene, not all would benefit from anti‐EGFR therapy.^8^ Similarly, for anti‐VEGF therapies, combination of bevacizumab and chemotherapy increased 10% response rate in metastatic colon cancer, from 34.8% in chemotherapy alone to 44.8% when combined with bevacizumab.^9^ Even with the combination of chemotherapy and targeted therapies, there are still a significant proportion of patients who failed to show any benefits. Those unmet medical needs urge scientists and industry to find new therapeutic targets which could help increase the survival rate of metastatic colon cancer patients.

Propofol is widely used as an intravenous sedative‐hypnotic agent in cancer resection surgery.^10^ Besides, recent studies even found that propofol also exerts antitumor activities in several types of cancers, such as breast cancer, lung cancer, pancreatic cancer, hepatocellular carcinoma, and colon cancer.^11^, ^12^, ^13^, ^14^, ^15^ Although some of the results remain controversial, overall, propofol has been considered to possesses an antitumor role on the proliferation, invasion, and metastasis of tumor cells via regulating key RNAs and signaling pathways.^10^ However, the underlying mechanisms about how propofol suppresses the development of colon cancer remain unclear.

Long noncoding RNAs (lncRNAs) are functional RNAs which do not encode proteins. It has been widely accepted that high expression of lncRNA HOTAIR (HOX anti‐sense intergenic RNA) was associated with poor survival in different types of tumors.^16^, ^17^, ^18^, ^19^ Besides, high HOTAIR levels also indicated poor prognosis of patients in colon cancer.^20^ In addition, HOTAIR expression was higher in colon cancer compared to normal tissue, which was closely related to liver metastasis.^21^ Moreover, HOTAIR was found to be involved in the antitumor effects of propofol in cervical cancer.^22^ However, whether propofol gets involved in modulating the tumor progression of colon cancer through inhibition of HOTAIR remains elusive.

The Wnt signaling pathway plays important roles in the carcinogenesis of several types of cancers, especially colon cancer,.^23^ HOTAIR was shown to activate Wnt pathway through inhibition of WIF‐1 expression,^17^ and could participate in the drug resistance.^24^ Furthermore, STAT3 (signaling transducer and activator of transcription 3) has been proved to regulate the activity of HOTAIR, and influence the biological functions of cervical cancer.^25^ In addition, STAT3 could also promote epithelial‐mesenchymal transition (EMT) in colon cancer, thus contributing to the cancer metastasis.^26^

Herein, our study investigates the underlying mechanisms of propofol on regulating the metastasis of colon cancer. We revealed that propofol could suppress the development and metastasis of colon cancer by blocking the interaction of STAT3 with HOTAIR promoter and downregulating the expression of HOTAIR, which causes the repression of Wnt signaling pathway via WIF‐1. Therefore, these findings further clarify the underlying mechanisms of propofol exerting anti‐tumor activities, suggesting that propofol might serve a novel treatment for colon cancer in the future.

## 2 METHODS

### 2.1 Cell culture

Human colon cancer cell lines (LOVO and SW480 cells) and normal human colon mucosal epithelial cell line (NCM460) were purchased from ATCC (American Type Culture Collection). After thawing, cells were transferred into flasks filled with DMEM (with 10% fetal bovine serum) (GIBCO) and cultured in Dulbecco's modified Eagle medium (DMEM; GIBCO) supplemented with 10% fetal bovine serum (FBS, GIBCO), penicillin (100 U/mL, Sigma), and streptomycin (100 μg/mL, Sigma). The cells were kept in a humidified atmosphere with 5% CO_2_ at 37°C. For propofol treatment, about 8 μg/mL concentration was used.

### 2.2 Plasmid construction and transfection

For loss‐of‐function analysis of HOTAIR or STAT3, the siRNAs sequences targeting human HOTAIR and STAT3 were designed and produced by GenePharma. For gain‐of‐function analysis of HOTAIR, the full length of HOTAIR was amplified from human cDNA and HOTAIR gene was inserted into pcDNA3.1 (Invitrogen) vectors. Then, cell transfection was performed using Lipofectamine 2000 (Invitrogen) according to the manufacturer's instructions.

### 2.3 Cell apoptosis assay

Cell apoptosis of colon cancer cell lines was determined using Annexin V‐propidium iodide (PI) kit (BD Biosicences) based on the manufacturer's instructions. Briefly, after washing and resuspending in binding buffer, cells were stained with Annexin V and PI for 15 minutes in dark. Apoptosis of the colon cancer cells were then measured using flow cytometry (Beckman).

### 2.4 Immunofluorescence staining

Sterile coverslips were put into wells of a six‐well plate and 0.1% gelatin was added into each well. After incubation for 10 minutes, coverslips were dried for 15 minutes and 500 μL cultured cells were added into each well at a concentration of 10^4^ per mL. After cells reached 80% confluence, culture media was removed and wells were washed with 1 × PBS. Cells were then fixed with 400 μL 4% formaldehyde for 10 minutes and washed with 1 × PBS. After being permeabilized by 0.5% Triton‐X 100 (Sigma) for 5 minutes and blocking nonspecific staining in 1 × PBS with 1% BSA (Sigma) and 1% goat serum (Sigma), cells were incubated overnight at 4°C with β‐catenin (1:200, ab32572, Abcam), E‐cadherin (1:100, ab1416, Abcam), or N‐cadherin (1:200, ab18203, Abcam) primary antibody, followed by incubation with fluorochrome‐conjugated secondary antibodies at room temperature for 1 hour. Slides were then washed with PBS and mounted with antifade mountants (Life Technologies). Result was obtained under fluorescence microscope (Olympus).

### 2.5 Cell invasion assessment

Transwell assay was carried out using modified Boyden chambers (Transwell). Briefly, after being starved in serum‐free medium (Sigma) for 24 hours, trypsinized, and resuspended in 1 × PBS, 3.0 × 10^5^, cells were placed in the upper chamber of transwells with Matrigel. Cells were then incubated at 37°C for 24 hours, and stained using 1% crystal violet. Viability of invasive cells was then evaluated.

### 2.6 Extraction of total RNA and quantitative real‐time PCR

Total RNA was extracted from cultured cells or tumor tissue using TRIzol Reagent (Invitrogen). The culture media were removed once the cells reached 80% confluence were directly lysed using 0.3 mL TRIzol Reagent. For tumor tissue, around 70 mg of tumor tissue was homogenized in 1 mL TRIzol reagent. Isopropanol was added into the homogenates and the mixture was incubated for 10 minutes and centrifuged at 12 000 × *g* for 10 minutes at 4°C. Supernatant was discarded and the RNA pellet was resuspended in 75% ethanol. After centrifugation at 7500 × *g* for 5 minutes at 4°C, supernatant was discarded and RNA pellet was air‐dried for 5 minutes. The RNA pellet was then resuspended in 20 μL of RNase‐free water with 0.1 mmol/L EDTA, and then incubated at 55°C for 15 minutes. RNA concentration was determined by measuring the ratio of absorbance at 260 and 280 nm. For reverse transcription and quantitative real‐time PCR, a SuperScript IV One‐Step RT‐PCR system (#12594100, ThermoFisher) was used as per the manufacturer's instruction. Briefly, 1 μg of RNA from each sample was mixed with 10 μmol/L forward primer, 10 μmol/L reverse primer, Master Mix, and RT Mix. Nuclease‐free water was added into the mixture to a final volume of 50 μL. Reverse transcription was performed at 60°C for 10 minutes and quantitative real‐time PCR was performed for 40 cycles on an Applied Biosystems 7500 (ABI). The relative expressions of genes were calculated using 2^−ΔΔCT^ method with normalization to GAPDH. Gene‐specific primers were as follows: HOTAIR forward: GGTAGAAAAAGCAACCACGAAGC, reverse: ACATAAACCTCTGTCTGTGAGTGCC; WIF‐1 forward: TCCAAACACCTCAAAATGCTATC, reverse: GAACCCATCAGGACACTCGC; STAT3 forward: TAGCAGGATGGCCCAATGGAATCA, reverse: AGCTGTCACTGTAGAGCTGATGGA; GAPDH forward: CCGGGAAACTGTGGCGTGATGG, reverse: AGGTGGAGGAGTGGGTGTCGCTGTT.

### 2.7 Extraction of total cell protein and western blot

Cells were first trypsinized and resuspended in HLB buffer with protease inhibitors. After homogenization, cells were mixed with 1× SDS lysis buffer and boiled. Equal amount of protein from each sample was then separated using electrophoresis and transferred into nitrocellulose membranes. Membranes were then incubated with 5% skimmed milk, followed by overnight incubation with β‐catenin primary antibody (1:500, ab32572, Abcam), STAT3 primary antibody (1:1000, ab119352, Abcam), WIF‐1 primary antibody (1:1000, ab186845, Abcam), E‐cadherin primary antibody (1:1000, ab1416, Abcam), N‐cadherin primary antibody (1:1000, ab18203, Abcam), or GAPDH primary antibody (1:2000, ab9485, Abcam). This was followed by incubation with secondary antibody (1:2000, ab6721 or ab97265, Abcam, USA) for 2 hours. The protein bands of interest were visualized by an ECL Advanced Western Blot Detection Kit.

### 2.8 Construction of colon cancer xenograft mice model

Twenty‐four male nude mice (4 weeks, 16 g) were purchased from the Nanjing Medical University, Animal Experiment Center and kept in SPF‐grade environment. All the experiments on the animals were approved by Zhengzhou University, Affiliated Cancer Hospital. Briefly, the animals were randomly allocated into one of the five groups (model, model + DMSO, model + Propofol, model + siHOTAIR, model + siNC, with n = 6 in each group) which were transplanted with colon cancer cells only (control), colon cancer cells and DMSO (i.p., once per week, 4 continuous weeks), colon cancer cells and propofol (i.p., once per week, 4 continuous weeks), or colon cancer cells transfected with siRNA targeting HOTAIR (siHOTAIR) or negative control (siNC), respectively. Colon cancer cell lines, LOVO or SW480 (5X10^6^ cells), or those transfected with siRNA targeting HOTAIR (siHOTAIR group) or negative control (siNC) were first injected subcutaneously. Tumor size was recorded every 5 days. Animals were euthanized using carbon dioxide on day 30. Tumor samples were quickly dissected from the animal and transferred to −80°C freezer to avoid possible degradation.

### 2.9 Immunohistochemistry

Tumor samples used for immunohistochemistry were fixed in 3.7% paraformaldehyde for 24 hours. After dehydration and paraffin embedding, tumor samples were sectioned. Before the staining, slides were deparaffinized and rehydrated in deionized water. Antigen retrieval was performed using pressure cooker. Briefly, slides were bathed in 1× Tris‐EDTA antigen retrieval buffer (ab93684, Abcam) and heated in pressure cooker with full pressure for 3 minutes. The pressure cooker and slides were then cooled down under running tap water. Slides were then incubated with WIF‐1 primary antibody (1:100, ab186845, Abcam) at 4°C overnight, followed by incubation with secondary antibody (1:1000, Santa Cruz, CA) for 2 hours at room temperature after which protein signals were visualized using diaminobenzidine. Control experiments without primary antibodies demonstrated that the signals observed were specific.

### 2.10 Luciferase reporter assay

The predicted binding sites of STAT3 in the HOTAIR promoter region was into the Kpnl and HindIII sites of the firefly luciferase in pGL3 vector (Promega). Then, LOVO and SW480 cells were cotransfected with the plasmids and siSTAT3 or its negative control (siNC) using Lipofectamine 2000 (Invitrogen), according to the manufacturer's instructions. About 48 hours after transfection, the relative luciferase activity was determined by the dual‐luciferase reporter assay system (Promega).

### 2.11 Statistical analysis

SPSS (Chicago) was used for the statistical analysis. All data are presented as mean ± standard deviation from three independent repeats. *Student's t* test and one‐way ANOVA were used to compare the differences between different groups. Results were statistically significant if *P* < .05.

## 3 RESULTS

### 3.1 Propofol induced the apoptosis and inhibited invasion of colon cancer cell lines

**Figure 1:**
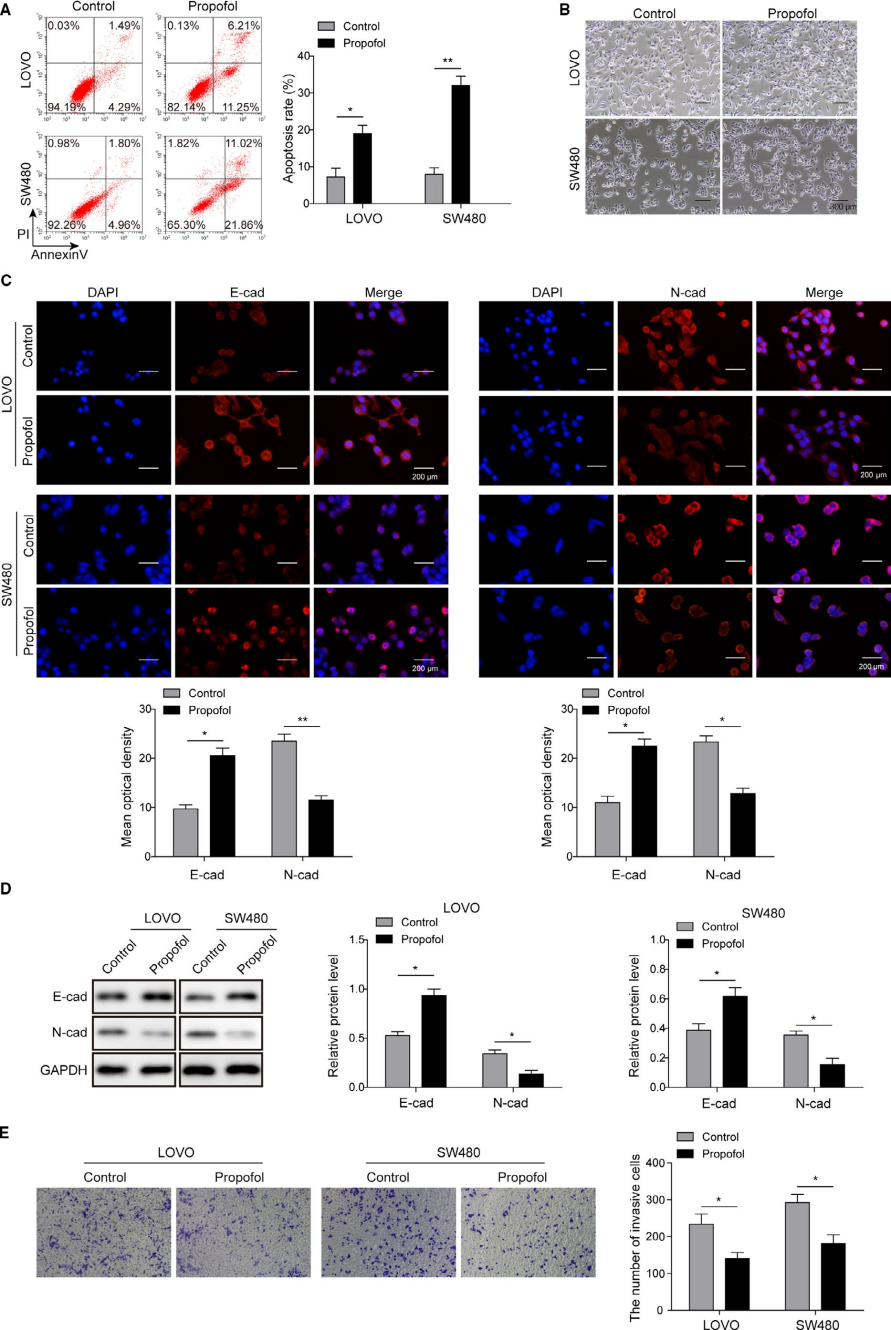
Propofol induced the apoptosis of colon cancer cell lines and inhibited their invasion. A, Flow cytometry showed that apoptotic rate of both LOVO and SW480 were significantly increased after propofol treatment; *P < .01 and **P < .05 vs Control. B, Propofol‐treated LOVO and SW480 cells showed less mesenchymal and more epithelial phenotype compared to control; Scale bar, 300 µm. C, Immunofluorescence staining was subjected to detect the expression of E‐cadherin and N‐cadherin in both LOVO and SW480 cells treated with propofol; Scale bar, 200 µm. *P < .01 and **P < .05 vs Control. D, Western blot analysis was performed to determine the expression of E‐cadherin and N‐cadherin in both LOVO and SW480 cells treated with propofol. *P < .05 vs Control. E, Transwell assay showed decreased invasive cell number after propofol treatment in both LOVO and SW480 cells. *P < .05 vs Control

LOVO and SW480 colon cancer cells were cultured and treated with 8 μg/mL propofol as described.^15^ After treatment, the cells showed significantly higher apoptotic rate compared to control (Figure 1A). Besides, propofol‐treated cells also showed less mesenchymal and more epithelial phenotype under microscope (Figure 1B). Consistently, the immunofluorescence staining results also showed more E‐cadherin expression and less N‐cadherin expression in propofol‐treated cells (Figure 1C), indicating that EMT was suppressed with the application of propofol in both colon cancer cell lines. Additionally, transwell assay presented that invasive cells was significantly reduced in propofol‐treated group compared to control (Figure 1E). Thus, these data suggested that propofol could induce apoptosis and suppress invasion of colon cancer cell lines.

### 3.2 Propofol regulated cell apoptosis and invasion via down‐regulating HOTAIR

**Figure 2:**
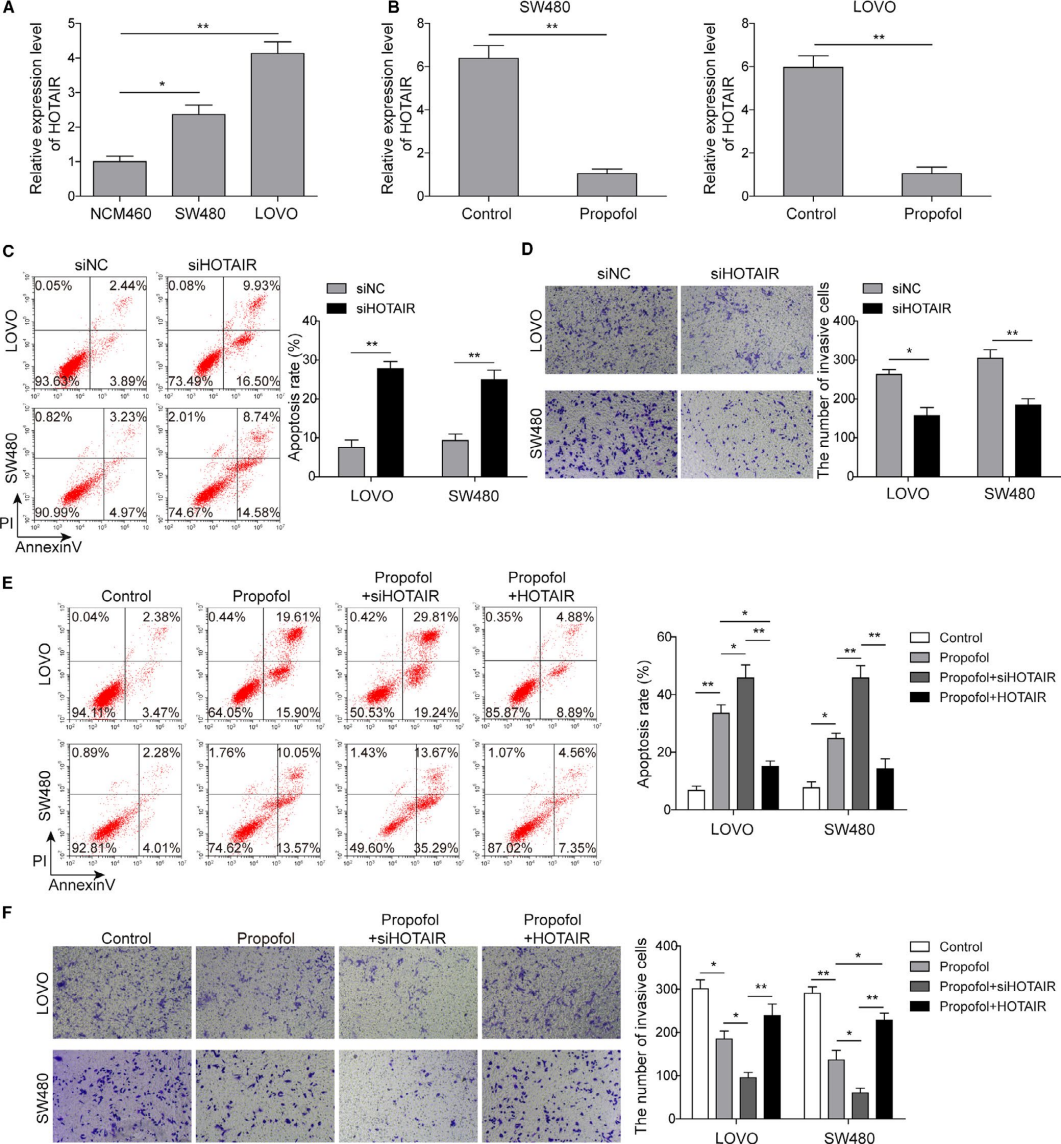
Propofol regulated cell apoptosis and invasion by downregulating lncRNA HOTAIR. A, Expression of lncRNA HOTAIR was significantly increased in colon cancer cells compared to normal intestinal epithelial cells; *P < .05 and **P < .01 vs NCM460. B, Propofol treatment significantly inhibited the expression of HOTAIR in both LOVO and SW480 cells; **P < .01 vs Control. C, Cell apoptosis was determined by the flow cytometry in both LOVO and SW480 cells transfected with siHOTAIR; **P < .01 vs siNC. D, Cell invasion was inhibited when HOTAIR expression was silenced by Transwell assay; *P < .05 and **P < .01 vs siNC. E, The flow cytometry was performed to measure the cell apoptosis in HOTAIR silenced or overexpression following propofol treatment; *P < .05 and **P < .01 vs Control. *P < .05 and **P < .01 vs Propofol. F, Transwell assay was performed to assess cell invasion in HOTAIR knocked‐down or overexpression with propofol treatment. *P < .05 and **P < .01 vs Control. *P < .05 and **P < .01 vs Propofol

To further explore the potential mechanism of propofol in cancer metastasis, qRT‐PCR was used to examine the expression of HOTAIR. First, the results showed that compared to control, colon cancer cells showed significantly higher expression of HOTAIR (Figure 2A). Moreover, HOTAIR expression was significantly repressed in colon cancer cells on propofol treatment (Figure 2B). In order to further investigate the role of HOTAIR, we then selectively knocked down HOTAIR expression in both LOVO and SW480 cell lines. Compared to the control group, HOTAIR knockdown group showed significantly higher apoptotic rate (Figure 2C) and less invasive cells (Figure 2D), indicating the oncogenic function of HOTAIR in colon cancer. To further verify whether there is the correlation between propofol and HOTAIR, RNA interference target HOTAIR and overexpression vector were transfected into colon cancer cells before exposure to propofol. The results showed that effects of propofol on apoptosis and invasion were reversed by HOTAIR overexpression, as indicated by significant decreased apoptotic cells and increased invasive cells, whereas the effects were further enhanced by HOTAIR knockdown (Figure 2E,F). These data indicated that propofol might regulate colon cancer cell apoptosis and invasion through regulation of HOTAIR.

### 3.3 HOTAIR expression was regulated by STAT3

**Figure 3:**
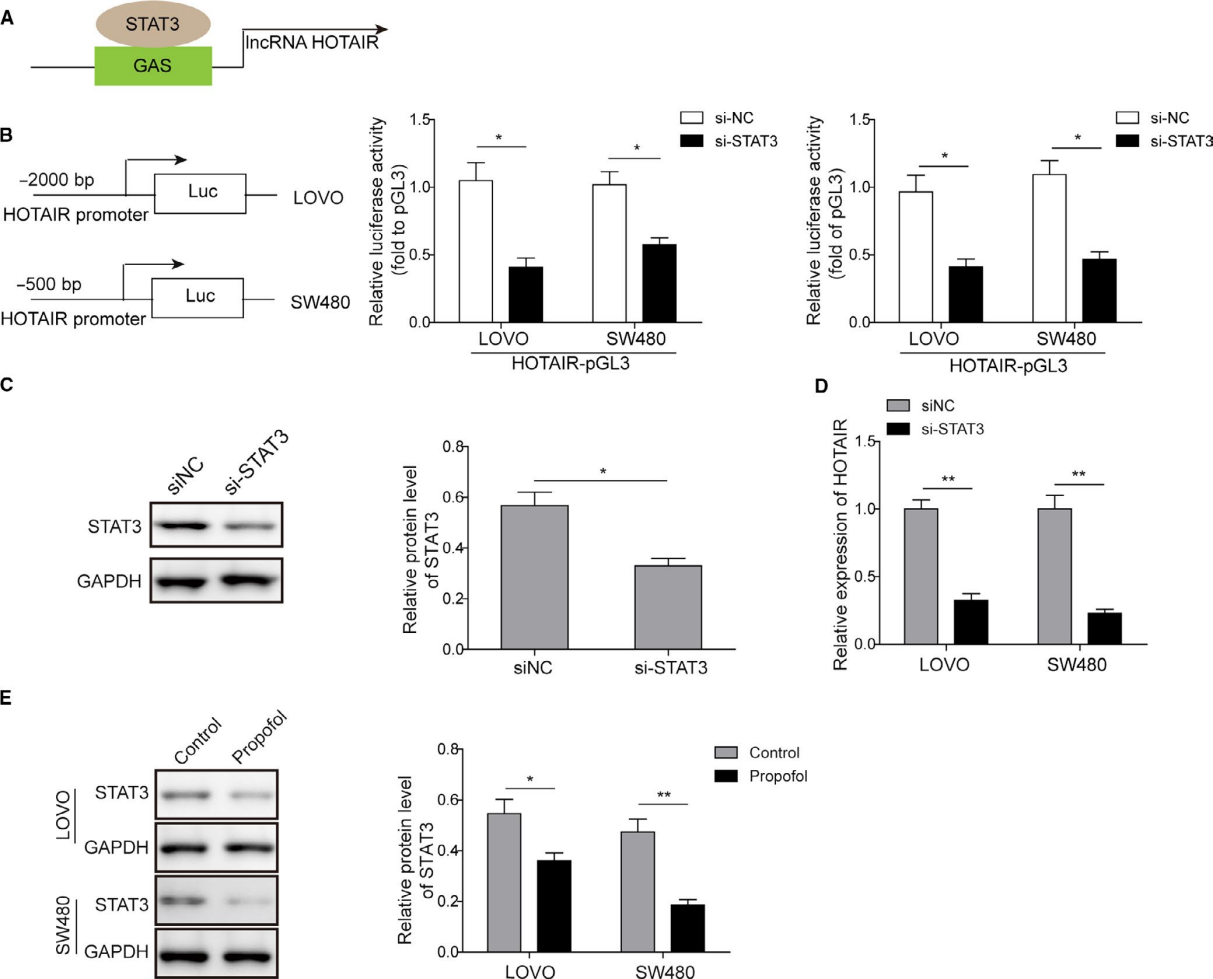
lncRNA HOTAIR expression was regulated by STAT3. A, Online analysis using MatInspector indicated the protein target of HOTAIR; B, Luciferase reporter assay was employed to test the regulation of STAT3 on HPTAIR promoter; *P < .05 vs siNC. C, Protein levels of STAT3 were decreased after transfection of siRNA targeting STAT3 (si‐STAT3) in colon cancer cells; *P < .05 vs siNC. D, Expression of HOTAIR was significantly decreased when STAT3 was silenced (si‐STAT3); **P < .01 vs siNC. E, Expression levels of STAT3 were decreased after propofol treatment in both LOVO and SW480 cell lines. *P < .05 and **P < .01 vs Control

Previous study has been reported that STAT3 could bind to HOTAIR promoter (GAS element), as indicated by MatInspector online software.^25^ We hereby validated this finding and the results confirmed a binding site for STAT3 on HOTAIR promoter (Figure 3A). Moreover, the dual‐luciferase reporter assay further verified that STAT3 could positively regulate the luciferase activity of HOTAIR promoter (Figure 3B). Using siRNA specifically targeting STAT3, we selectively silenced STAT3 expression in colon cancer cells, which was certified using western blot (Figure 3C). Subsequently, qRT‐PCR analysis also showed that silencing STAT3 significantly induced downregulation of HOTAIR (Figure 3D), suggesting that HOTAIR expression was positively regulated by STAT3. Additionally, propofol could also cause STAT3 downregulation (Figure 3E), suggesting that propofol may inhibit HOTAIR expression via STAT3.

### 3.4 HOTAIR and STAT3 independently regulated colon cancer cell apoptosis and invasion

**Figure 4:**
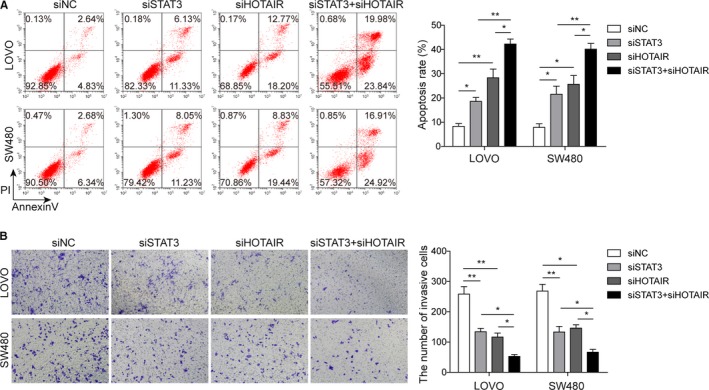
STAT and HOTAIR independently regulate the apoptosis and invasion of colon cancer cells. A, Silencing of either STAT3 (siSTAT3) or HOTAIR (siHOTAIR) increased the apoptosis rate of colon cancer cells, while silencing both STAT3 and HOTAIR (siSTAT3 + siHOTAIR) showed the highest apoptosis rate among the four groups; *P < .05 and **P < .01 vs siNC. *P < .05 and **P < .01 vs siSTAT3 + siHOTAIR. B, Similarly, silencing of either STAT3 (siSTAT3) or HOTAIR (siHOTAIR) led to decreased invaded cells in Transwell assay, and silencing both STAT3 and HOTAIR (siSTAT3 + siHOTAIR) showed the least invaded cells among the four groups; *P < .05 and **P < .01 vs siNC. *P < .05 vs siSTAT3 + siHOTAIR

For further verification of the correlation between HOTAIR and STAT3, RNA interference for silencing both HOTAIR and STAT3 was applied. As shown in Figure 4A, selective silencing of HOTAIR or STAT3 led to an increase in cell apoptotic rate. Interestingly, when we silenced both HOTAIR and STAT3 in colon cancer cells, the apoptotic rate was higher than knockdown on either HOTAIR or STAT3 alone (Figure 4A). Similarly, silencing of both HOTAIR and STAT3 also showed the largest inhibition on cell invasion abilities, compared to HOTAIR or STAT3 alone (Figure 4B). Those results indicated that STAT3 and HOTAIR could independently promote colon cancer cell apoptosis and inhibit cell invasion.

### 3.5 HOTAIR promoted cells invasion through WIF‐1 and Wnt signaling pathway

**Figure 5:**
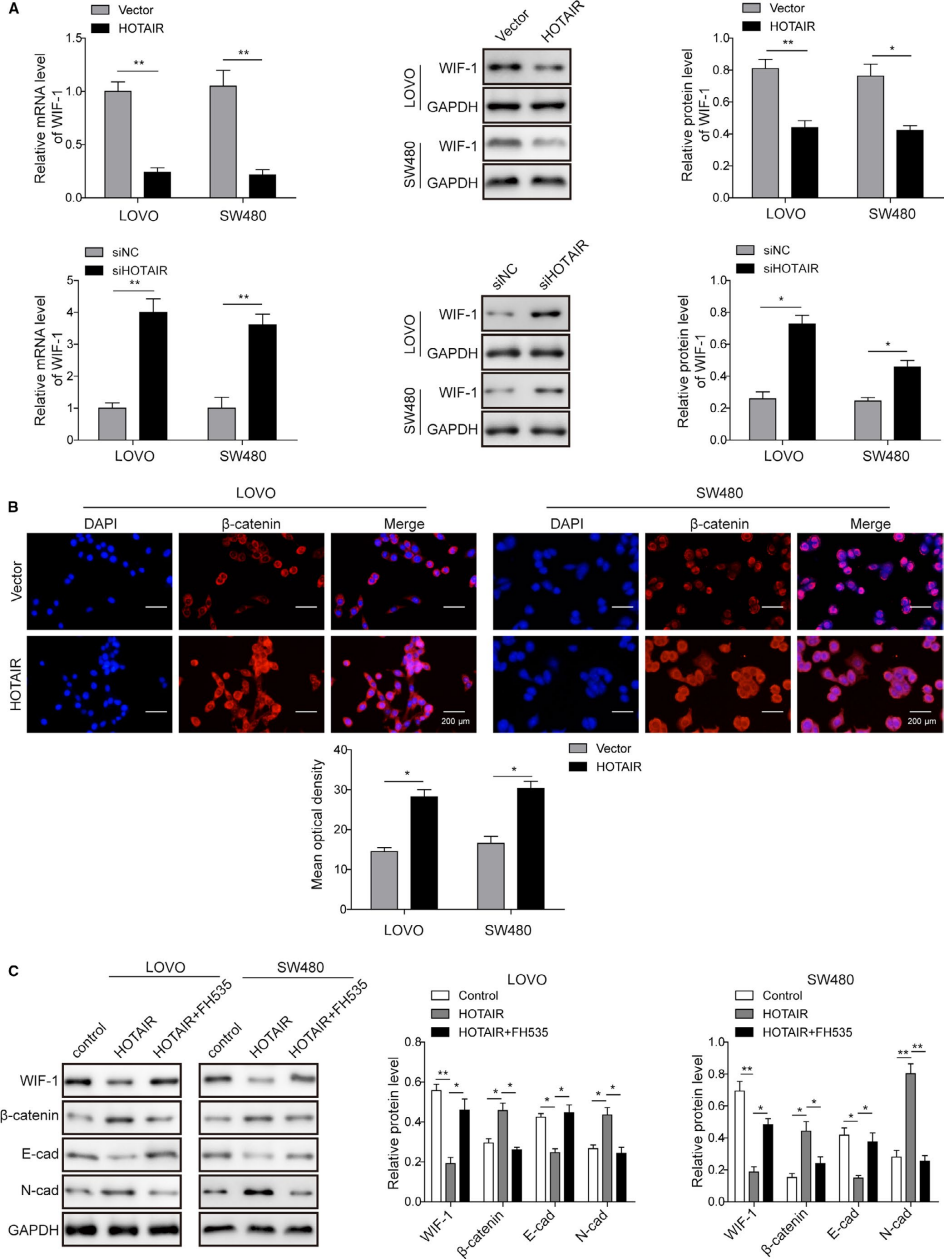
HOTAIR promoted cell invasion through inhibiting WIF‐1 expression and activating Wnt signaling pathway. A, Overexpression of HOTAIR decreased WIF‐1 expression, and silencing of HOTAIR increased WIF‐1 expression in both mRNA and protein levels in LOVO and SW480; *P < .05 and **P < .01 vs Vector. *P < .05 and *P < .05 and **P < .01 vs siNC. (B) Overexpression of HOTAIR promoted the nuclear accumulation of β‐catenin in both LOVO and SW480 cell lines; Scale bar, 200 µm. (C) HOTAIR overexpression (HOTAIR) decreased E‐cadherin expression and increased N‐cadherin expression levels; while the effect was reversed by the application of FH535. *P < .05 and **P < .01 vs Control. *P < .05 and **P < .01 vs HOTAIR

HOTAIR has been found to activate Wnt signaling pathway via inhibition of WIF‐1 expression in esophageal squamous cell carcinoma.^17^ In this study, we also investigated the possible activation of Wnt signaling pathway in colon cancer. After silencing HOTAIR expression in LOVO and SW480 cells, WIF‐1 expression was increased (Figure 5A), while the opposite trend was observed within HOTAIR overexpression (Figure 5A). Furthermore, as a key factor in Wnt signaling pathway, nuclear accumulation of β‐catenin was also obviously increased when HOTAIR was overexpressed in both colon cancer cell lines (Figure 5B), indicating that HOTAIR enhanced the activation of Wnt pathway. Further analysis on the EMT‐related markers showed that HOTAIR overexpression inhibited E‐cadherin expression and increased N‐cadherin level (Figure 5C), indicating that HOTAIR could promote EMT in colon cancer cell lines. However, treatment of FH535, an inhibitor for Wnt signaling pathway,^27^ reversed the effects of HOTAIR overexpression on β‐catenin, E‐cadherin, N‐cadherin, and WIF‐1 (Figure 5C).

### 3.6 Propofol inhibited tumor growth of colon cancer growth in vivo

**Figure 6:**
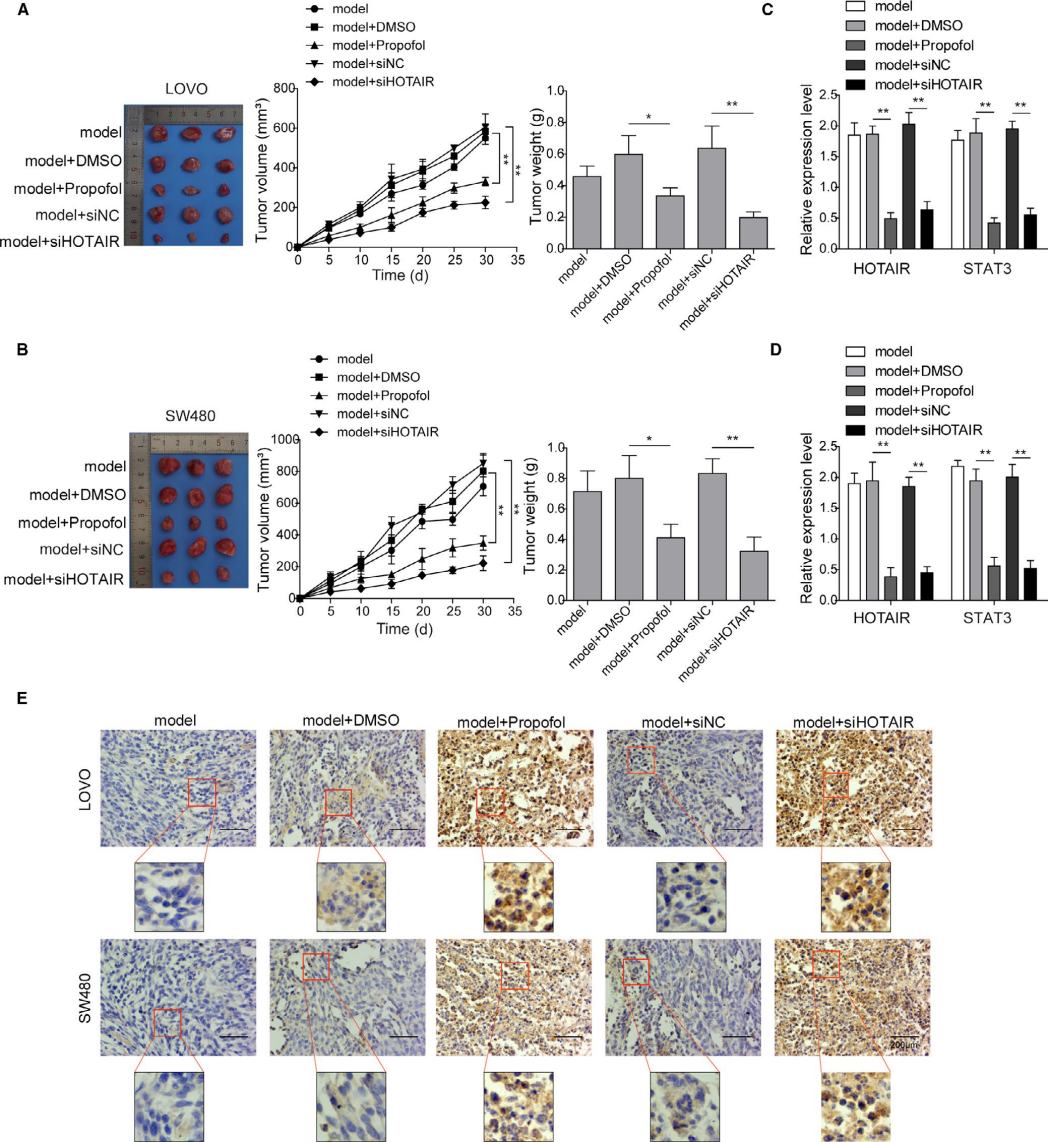
Propofol inhibited the tumor progression of colon cancer in vivo. Nude mice were divided into four groups which were given colon cancer cell transplantation only (model), colon cancer cells and DMSO (model + DMSO), colon cancer cells and propofol (model + propofol), colon cancer cells transfected with siRNA targeting HOTAIR (model + siHOTAIR) or its negative control (model + siNC), respectively. (A) and (B) In xenograft model using LOVO and SW480 cells, tumor volume and weight, (C) and (D) expression levels of STAT3 and HOTAIR were significantly reduced in model + propofol or model + siHOTAIR groups compared to model and model + DMSO groups; **P < .01 vs model. (E) Expression of WIF‐1 was significantly elevated in model + propofol and model + siHOTAIR groups compared to model and model + DMSO groups. Scale bar, 200 µm

BALB/C nude mice was performed to establish xenograft tumor colon cancer model. Tumor volume was significantly lower in the propofol or siHOTAIR group, compared with control groups (model group, model + DMSO, model + siNC group) (Figure 6A,B). In addition, mRNA expression of STAT3 and HOTAIR was also significantly decreased in propofol and siHOTAIR groups (Figure 6C,D). Meanwhile, immunohistochemistry staining revealed that WIF‐1 expression was enhanced in propofol and siHOTAIR groups (Figure 6E).Overall, these findings suggest that propofol or HOTAIR silencing inhibited tumor growth. Propofol may exert similar inhibitory effect on in vivo tumor metastasis, and this effect was probably due to the reduced expression of STAT3, HOTAIR, and WIF‐1 expression and subsequent activation of Wnt signaling pathway.

## 4 DISCUSSION

Propofol is a widely used sedative agent in cancer resection surgery. Recently, propofol was proved to exert antitumor activities in several types of cancers. Previous study revealed that propofol could inhibit cancer growth and invasion through Wnt/β‐catenin pathway.^11^ Through inducing endoplasmic reticulum stress, propofol increased cell apoptosis in lung cancer.^13^ Moreover, propofol could also suppress the pancreatic cancer proliferation and invasion via microRNA‐133a.^14^ Besides, propofol has also been reported to inhibit the colon cancer invasion, via downregulation of MMP‐2 and MMP‐9.^15^ Herein, in our study, we also observed that propofol induced apoptosis of colon cancer cells and inhibited cell invasion, by regulating HOTAIR expression levels.

It has been widely recognized that HOTAIR plays critical roles in cancer cell proliferation, apoptosis, invasion, metastasis, etc.^28^ Expression level of HOTAIR was higher in colon cancer tissue and blood of patients, and was related to higher mortality.^20^, ^21^ In addition, HOTAIR was a predictor of metastasis in colon cancer, while the underlying mechanism involved inhibiting E‐cadherin and increasing MMP‐9 and vimentin expressions.^29^ Similar to those previous findings, our study showed that knockdown of HOTAIR promoted cell apoptosis and inhibited cell invasion, while regulation on HOTAIR expression levels could influence propofol's antitumor effect. Further mechanistic study also demonstrated that propofol could exert its anti‐tumor activity through inhibition of HOTAIR expression.

STAT3 plays an important role in cancer cell proliferation and invasion, which is intensively studied as a new therapeutic target.^30^, ^31^ Multiple regulation pathways (eg, EGFR, VEGF pathways) were involved in the activation of STAT3 during tumor progression, which influence the EMT, invasion, and metastasis of cancer cells and change tumor microenvironment.^32^ STAT3 was also found to regulate HOTAIR the expression, thus contributing to the progression of cancers.^25^, ^33^ Similarly, our study showed that STAT3 could also regulate HOTAIR expression in colon cancer, and in addition, propofol could decrease STAT3 expression, which then contributed to the inhibition of HOTAIR expression during propofol treatment. In colon cancer, STAT3 could regulate the EMT in a ZEB1‐dependent pathway, where knockdown of ZEB1 blocked the promoting effects of STAT3 on the EMT colon cancer cell.^26^ Unlike the relationship between STAT3 and ZEB1, our study showed that HOTAIR and STAT3 independently regulate colon cancer cell apoptosis and invasion.

Upon its activation, lncRNA HOTAIR could activate several downstream signaling pathways, including predominant polycomb repressive complex 2 component (EZH2),^19^ polycomb‐dependent chromatin modification,^21^ and Wnt signaling pathway *via* WIF‐1.^17^, ^34^ As a hallmark of colon cancer, activation of Wnt signaling pathway was important in the tumorigenesis and metastasis of colon cancer.^23^, ^35^ As the key event in the activation of Wnt signaling pathway, β‐catenin nuclear localization was enhanced in the invasive edge of tumor tissue, implying EMT in those cells.^35^ In our study, we also observed that lncRNA HOTAIR could activate Wnt signaling pathway by inhibiting WIF‐1, which indicates that propofol's inhibitory effect on colon cancer invasion and metastasis might be via lncRNA HOTAIR inhibition and subsequent deactivation of Wnt signaling pathway. In addition, these results indicate that propofol might be a potential treatment for colon cancer with overactivated Wnt signaling.

We then further validated the above findings using the xenograft tumor colon cancer model in vivo. Xenograft mice model is widely used in the investigation of the underlying mechanism of tumorigenesis and drug responses of colon cancer.^36^, ^37^, ^38^, ^39^, ^40^ Our results showed that propofol treatment or knockdown of lncRNA HOTAIR decreased tumor volume and weight, and inhibited expression of STAT3 and lncRNA HOTAIR, while WIF‐1 expression was enhanced. These results further support our in vitro findings described above.

## 5 CONCLUSION

Taken together, our results revealed that propofol could promote apoptosis and inhibit invasion, mainly through its inhibition on STAT3 and HOTAIR to deactivate Wnt signaling pathway by increasing WIF‐1 expression levels, which might serve as a therapeutic role for colon cancer. Our study, however, did not investigate the effect of propofol in patients diagnosed with colon cancer and undergoing surgical surgery, which may help to further testify the function of propofol on colon cancer. Future studies may involve larger sample size and more detailed methods. Further investigation is also required to understand the relationship between lncRNA HOTAIR and roles of ERK1/2 and MMPs in the antitumor activities of propofol.

## CONFLICT OF INTEREST

The authors declare that they have no conflict of interest.

## Data Availability

All data generated or analysed during this study are included in this published article.

## References

[1] Brenner H , Kloor M , Pox CP . Colorectal cancer. Lancet. 2014;383(9927):1490‐1502. 24225001 10.1016/S0140-6736(13)61649-9

[2] van de Velde CJ , Boelens PG , Borras JM , et al, EURECCA colorectal: multidisciplinary management: European consensus conference colon & rectum. Eur J Cancer, 2014;50(1):1 e1‐1 e34. 24183379 10.1016/j.ejca.2013.06.048

[3] Kuipers EJ , Grady WM , Lieberman D , et al. Colorectal cancer. Nat Rev Dis Primers. 2015;1:15065. 27189416 10.1038/nrdp.2015.65 PMC4874655

[4] Saltz LB , Meropol NJ , Loehrer PJ , Needle MN , Kopit J , Mayer RJ . Phase II trial of cetuximab in patients with refractory colorectal cancer that expresses the epidermal growth factor receptor. J Clin Oncol. 2004;22(7):1201‐1208. 14993230 10.1200/JCO.2004.10.182

[5] Van Cutsem E , Peeters M , Siena S , et al. Open‐label phase III trial of panitumumab plus best supportive care compared with best supportive care alone in patients with chemotherapy‐refractory metastatic colorectal cancer. J Clin Oncol. 2007;25(13):1658‐1664. 17470858 10.1200/JCO.2006.08.1620

[6] Amado RG , Wolf M , Peeters M , et al. Wild‐type KRAS is required for panitumumab efficacy in patients with metastatic colorectal cancer. J Clin Oncol. 2008;26(10):1626‐1634. 18316791 10.1200/JCO.2007.14.7116

[7] Karapetis CS , Khambata‐Ford S , Jonker DJ , et al. K‐ras mutations and benefit from cetuximab in advanced colorectal cancer. N Engl J Med. 2008;359(17):1757‐1765. 18946061 10.1056/NEJMoa0804385

[8] Misale S , Di Nicolantonio F , Sartore‐Bianchi A , Siena S , Bardelli A . Resistance to anti‐EGFR therapy in colorectal cancer: from heterogeneity to convergent evolution. Cancer Discov. 2014;4(11):1269‐1280. 25293556 10.1158/2159-8290.CD-14-0462

[9] Hurwitz H , Fehrenbacher L , Novotny W , et al. Bevacizumab plus irinotecan, fluorouracil, and leucovorin for metastatic colorectal cancer. N Engl J Med. 2004;350(23):2335‐2342. 15175435 10.1056/NEJMoa032691

[10] Jiang S , Liu YA , Huang L , Zhang F , Kang R . Effects of propofol on cancer development and chemotherapy: Potential mechanisms. Eur J Pharmacol. 2018;831:46‐51. 29654781 10.1016/j.ejphar.2018.04.009

[11] Ou W , Lv J , Zou X , et al. Propofol inhibits hepatocellular carcinoma growth and invasion through the HMGA2‐mediated Wnt/beta‐catenin pathway. Exp Ther Med. 2017;13(5):2501‐2506. 28565871 10.3892/etm.2017.4253 PMC5443290

[12] Ecimovic P , Murray D , Doran P , Buggy DJ . Propofol and bupivacaine in breast cancer cell function in vitro ‐ role of the NET1 gene. Anticancer Res. 2014;34(3):1321‐1331. 24596379

[13] Cui W‐Y , Liu Y , Zhu Y‐Q , Song T , Wang Q‐S . Propofol induces endoplasmic reticulum (ER) stress and apoptosis in lung cancer cell H460. Tumour Biol. 2014;35(6):5213‐5217. 24510348 10.1007/s13277-014-1677-7

[14] Wang ZT , Gong HY , Zheng F , Liu DJ , Dong TL . Propofol suppresses proliferation and invasion of pancreatic cancer cells by upregulating microRNA‐133a expression. Genet Mol Res. 2015;14(3):7529‐7537. 26214431 10.4238/2015.July.3.28

[15] Miao Y , Zhang Y , Wan H , Chen L , Wang F . GABA‐receptor agonist, propofol inhibits invasion of colon carcinoma cells. Biomed Pharmacother. 2010;64(9):583‐588. 20888181 10.1016/j.biopha.2010.03.006

[16] Bhan A , Mandal SS . LncRNA HOTAIR: a master regulator of chromatin dynamics and cancer. Biochim Biophys Acta. 2015;1856(1):151‐164. 26208723 10.1016/j.bbcan.2015.07.001 PMC4544839

[17] Ge X‐S , Ma H‐J , Zheng X‐H , et al. HOTAIR, a prognostic factor in esophageal squamous cell carcinoma, inhibits WIF‐1 expression and activates Wnt pathway. Cancer Sci. 2013;104(12):1675‐1682. 24118380 10.1111/cas.12296 PMC7653522

[18] Sørensen KP , Thomassen M , Tan Q , et al. Long non‐coding RNA HOTAIR is an independent prognostic marker of metastasis in estrogen receptor‐positive primary breast cancer. Breast Cancer Res Treat. 2013;142(3):529‐536. 24258260 10.1007/s10549-013-2776-7

[19] Zhang K , Sun X , Zhou X , et al. Long non‐coding RNA HOTAIR promotes glioblastoma cell cycle progression in an EZH2 dependent manner. Oncotarget. 2015;6(1):537‐546. 25428914 10.18632/oncotarget.2681 PMC4381613

[20] Svoboda M , Slyskova J , Schneiderova M , et al. HOTAIR long non‐coding RNA is a negative prognostic factor not only in primary tumors, but also in the blood of colorectal cancer patients. Carcinogenesis. 2014;35(7):1510‐1515. 24583926 10.1093/carcin/bgu055

[21] Kogo R , Shimamura T , Mimori K , et al. Long noncoding RNA HOTAIR regulates polycomb‐dependent chromatin modification and is associated with poor prognosis in colorectal cancers. Cancer Res. 2011;71(20):6320‐6326. 21862635 10.1158/0008-5472.CAN-11-1021

[22] Zhang D , Zhou X‐H , Zhang J , et al. Propofol promotes cell apoptosis via inhibiting HOTAIR mediated mTOR pathway in cervical cancer. Biochem Biophys Res Commun. 2015;468(4):561‐567. 26523512 10.1016/j.bbrc.2015.10.129

[23] Sebio A , Kahn M , Lenz HJ . The potential of targeting Wnt/beta‐catenin in colon cancer. Expert Opin Ther Targets. 2014;18(6):611‐615. 24702624 10.1517/14728222.2014.906580

[24] Guo F , Cao Z , Guo H , Li S . The action mechanism of lncRNA‐HOTAIR on the drug resistance of non‐small cell lung cancer by regulating Wnt signaling pathway. Exp Ther Med. 2018;15(6):4885‐4889. 29805510 10.3892/etm.2018.6052 PMC5958754

[25] Zhang Y , Cheng X , Liang H , Jin Z . Long non‐coding RNA HOTAIR and STAT3 synergistically regulate the cervical cancer cell migration and invasion. Chem Biol Interact. 2018;286:106‐110. 29572071 10.1016/j.cbi.2018.03.010

[26] Xiong H , Hong J , Du W , et al. Roles of STAT3 and ZEB1 proteins in E‐cadherin down‐regulation and human colorectal cancer epithelial‐mesenchymal transition. J Biol Chem. 2012;287(8):5819‐5832. 22205702 10.1074/jbc.M111.295964 PMC3285352

[27] Liu J , Li G , Liu D , Liu J . FH535 inhibits the proliferation of HepG2 cells via downregulation of the Wnt/beta‐catenin signaling pathway. Mol Med Rep. 2014;9(4):1289‐1292. 24482011 10.3892/mmr.2014.1928

[28] Tang Q , Hann SS . HOTAIR: an oncogenic long non‐coding RNA in human cancer. Cell Physiol Biochem. 2018;47(3):893‐913. 29843138 10.1159/000490131

[29] Wu Z‐H , Wang X‐L , Tang H‐M , et al. Long non‐coding RNA HOTAIR is a powerful predictor of metastasis and poor prognosis and is associated with epithelial‐mesenchymal transition in colon cancer. Oncol Rep. 2014;32(1):395‐402. 24840737 10.3892/or.2014.3186

[30] Yu H , Lee H , Herrmann A , Buettner R , Jove R . Revisiting STAT3 signalling in cancer: new and unexpected biological functions. Nat Rev Cancer. 2014;14(11):736‐746. 25342631 10.1038/nrc3818

[31] Wang SW , Sun YM . The IL‐6/JAK/STAT3 pathway: potential therapeutic strategies in treating colorectal cancer (Review). Int J Oncol. 2014;44(4):1032‐1040. 24430672 10.3892/ijo.2014.2259

[32] Yuan J , Zhang F , Niu R . Multiple regulation pathways and pivotal biological functions of STAT3 in cancer. Sci Rep. 2015;5:17663. 26631279 10.1038/srep17663 PMC4668392

[33] Sun S , Wu Y , Guo W , et al. STAT3/HOTAIR signaling axis regulates HNSCC growth in an EZH2‐dependent manner. Clin Cancer Res. 2018;24(11):2665‐2677. 29540490 10.1158/1078-0432.CCR-16-2248

[34] Cheng C , Qin Y , Zhi Q , Wang J , Qin C . Knockdown of long non‐coding RNA HOTAIR inhibits cisplatin resistance of gastric cancer cells through inhibiting the PI3K/Akt and Wnt/beta‐catenin signaling pathways by up‐regulating miR‐34a. Int J Biol Macromol. 2018;107(Pt B):2620‐2629. 29080815 10.1016/j.ijbiomac.2017.10.154

[35] Basu S , Haase G , Ben‐Ze'ev A . Wnt signaling in cancer stem cells and colon cancer metastasis. F1000Research. 2016;5:699. 10.12688/f1000research.7579.1 PMC4841194 27134739

[36] Chen EC , Karl TA , Kalisky T , et al. signaling promotes growth of colon xenograft tumors in mice and is up‐regulated in a subset of human colon cancers. Gastroenterology. 2015;149(3):705‐17 e2. 26026391 10.1053/j.gastro.2015.05.042 PMC4550533

[37] Li S , Yang G , Zhu X , Cheng L , Sun Y , Zhao Z . Combination of rapamycin and garlic‐derived S‐allylmercaptocysteine induces colon cancer cell apoptosis and suppresses tumor growth in xenograft nude mice through autophagy/p62/Nrf2 pathway. Oncol Rep. 2017;38(3):1637‐1644. 28737825 10.3892/or.2017.5849

[38] Bellamkonda K , Chandrashekar NK , Osman J , Selvanesan BC , Savari S , Sjölander A . The eicosanoids leukotriene D4 and prostaglandin E2 promote the tumorigenicity of colon cancer‐initiating cells in a xenograft mouse model. BMC Cancer. 2016;16:425. 27388564 10.1186/s12885-016-2466-z PMC4937611

[39] Liang G , Zhu Y , Jing A , et al. Cationic microRNA‐delivering nanocarriers for efficient treatment of colon carcinoma in xenograft model. Gene Ther. 2016;23(12):829‐838. 27482839 10.1038/gt.2016.60

[40] Wang J , Chen C , Wang S , et al. Bufalin inhibits HCT116 colon cancer cells and its orthotopic xenograft tumor in mice model through genes related to apoptotic and PTEN/AKT pathways. Gastroenterol Res Pract. 2015;2015:457193. 26770191 10.1155/2015/457193 PMC4685085

