# Supplementary figures and images for: Effects of propofol on colon cancer metastasis through STAT3/HOTAIR axis by activating WIF‐1 and suppressing Wnt pathway
Source: Cancer Med. 2020 Jan 17;9(5):1842–54. doi: 10.1002/cam4.2840 (PMC7050102; doi:10.1002/cam4.2840)

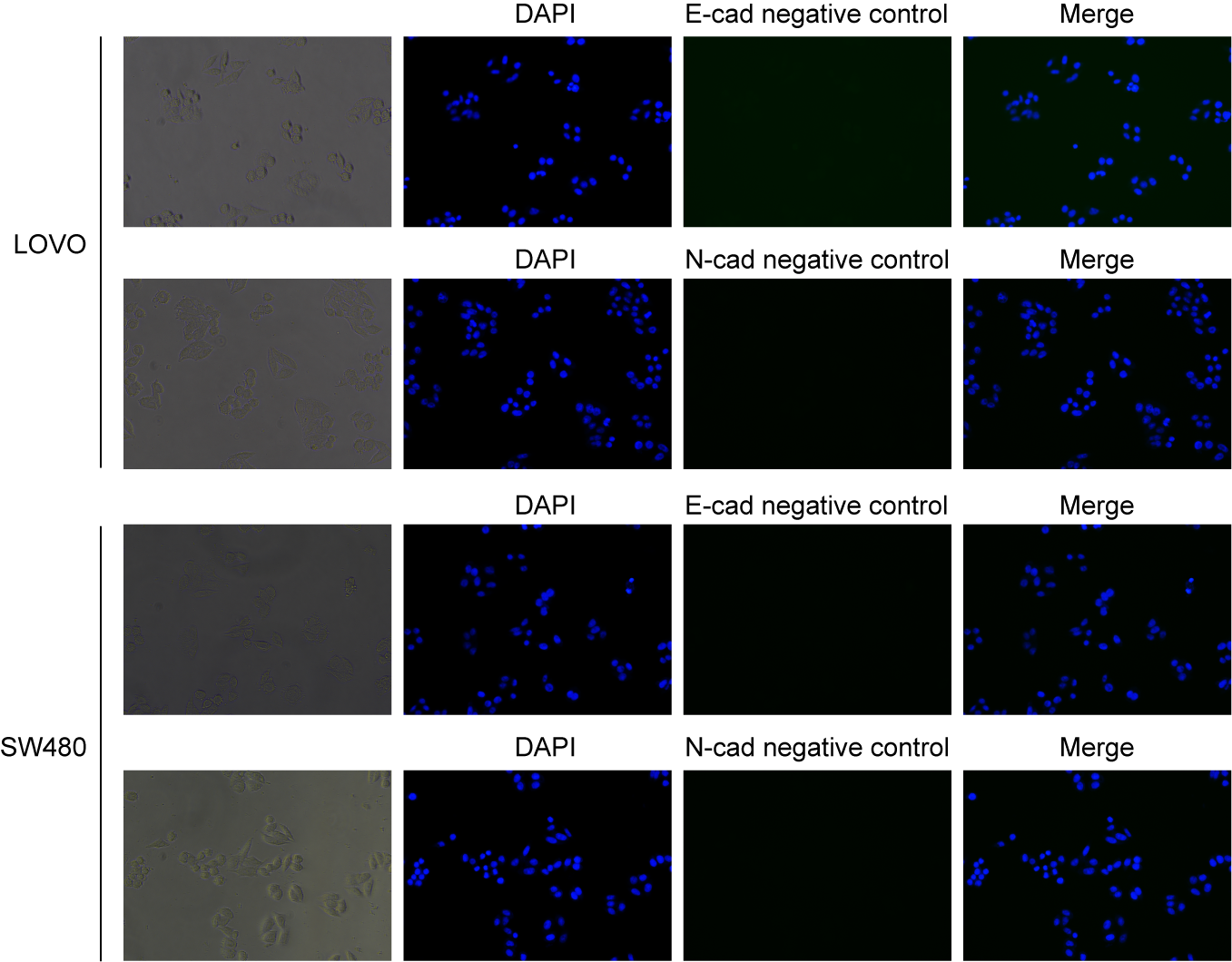

Supplement: Supplementary file 1 [file CAM4-9-1842-s001.tif]
